# Novel Spectroscopic Studies of the Interaction of Three Different Types of Iron Oxide Nanoparticles with Albumin

**DOI:** 10.3390/nano14231861

**Published:** 2024-11-21

**Authors:** Silviya Abarova, Tsenka Grancharova, Plamen Zagorchev, Boris Tenchov, Bissera Pilicheva

**Affiliations:** 1 Department of Medical Physics and Biophysics, Faculty of Medicine, Medical University of Sofia, 1000 Sofia, Bulgaria; silviya.m.abarova@gmail.com (S.A.); btenchov@gmail.com (B.T.); 2 Department of Medical Physics and Biophysics, Faculty of Pharmacy, Medical University of Plovdiv, 4002 Plovdiv, Bulgaria; tsenka.grancharova@mu-plovdiv.bg (T.G.); plamen.zagorchev@mu-plovdiv.bg (P.Z.); 3 Research Institute, Medical University of Plovdiv, 4002 Plovdiv, Bulgaria; 4 Department of Pharmaceutical Sciences, Faculty of Pharmacy, Medical University of Plovdiv, 4002 Plovdiv, Bulgaria

**Keywords:** iron oxide, nanoparticles, human serum albumin, fluorescence spectroscopy, UV-Vis spectroscopy, quenching, NPs binding

## Abstract

In the present work, we studied the interactions of three types of iron oxide nanoparticles (IONPs) with human serum albumin (HSA) by fluorescence and UV-Vis spectroscopy. The determined binding parameters of the reactions and the thermodynamic parameters, including ΔHo, ΔSo, and ΔGo indicated that electrostatic forces play a major role in the interaction of IONPs with HSA. These measurements indicate a fluorescent quenching mechanism based on IONPs-HSA static complex formation. Our study shows that the interaction between HSA and IONPs depends on the nanoparticle structure. The interaction between IONPs and HSA was found to be spontaneous, exothermic, and entropy-driven. HSA was shown to interact moderately with IONPs obtained with plant extracts of *Uncaria tomentosa* L. (IONP@UT) and *Clinopodium vulgare* L. (IONP@CV), and firmly with IONPs prepared with *Ganoderma lingzhi* (Reishi) extract (IONP@GL), via ground-state association. Analysis by modified Stern-Volmer approximation indicates that the quenching mechanism is static. Our study significantly improves our understanding of the mechanisms of interaction, distribution, and transport involved in the interaction between proteins and IONPs. It provides crucial insights into the functional perturbations of albumin binding capacity and the effects of IONPs on the stability and structural modifications of plasma carrier proteins.

## 1. Introduction

Because of their unique inherent properties, the most important of which is their small particle size, nanoparticles (NPs) can enhance interactions between biomolecules, creating novel NP–biological structures. On the particles’ surface, thermodynamic exchanges occur through dynamic interactions with biomolecules such as nucleic acids, enzymes, and proteins [1,2,3,4]. Thus, NP size, coating, and intrinsic physicochemical properties must be considered, as they are influenced by the combination of each component of the hybrid structure [5]. Studies have shown that NPs can alter drug-drug interactions and induce various effects on their pharmacodynamics and pharmacokinetics [6]. As many pharmacological agents bind extensively to plasma albumin, understanding how this process is modulated in the presence of NPs (protein-displacing interactions) is crucial. Such studies have been extensively performed worldwide [7]. New studies show that nanomaterials provide novel approaches to diagnosing and treating many diseases. When in the body, nanoparticles interact with biological molecules, most notably albumin, which can negatively affect the biological action of nanoparticles [8,9,10].

In this work, we synthesized nanosized iron oxide structures with potential therapeutic applications, consisting of an iron oxide core covered by a coating of plant extracts. The structural features of the IONPs were consistent with their interaction with albumin, a major blood protein, to demonstrate the effect of its binding on NP properties and protein retention. The thermodynamic parameters of the interaction between NPs and albumin were determined. We observed that IONPs coated with *Uncaria tomentosa* L. and *Clinopodium vulgare* L. extracts (IONP@UT and IONP@CV) showed lower affinity for albumin than those coated with Reishi extract (IONP@GL). We hypothesized that surface properties are crucial for the interaction between NPs and biomolecules. This study is the first to demonstrate a correlation between the structural properties of complex NPs coated with these natural substances and their interaction with albumin. However, such complex nanomaterials are expected to interact with biological molecules or cells once they enter the body. This may lead to various effects on the nanomaterial’s biodistribution and bioactivity [11,12]. It is well known that these interactions are influenced by the surface charge of the NPs, their size, shape, composition, hydrophobicity, and the binding affinity of biomolecules to their surface [13].

How the surface modification of NPs affects their biological activity has already been presented as follows: (i) pegylated NPs exhibit increased circulation time, avoiding clearance by the reticuloendothelial system; (ii) ligand-conjugated NPs show shorter circulation times but accumulate in targeted organs; (iii) “corona”-coated NPs allow prolonged circulation half-life and high targeting capacity [14]. When nanoparticles are introduced into biological fluids, they interact with components such as proteins, lipids, and sugars, forming a “corona” of biomolecules on their surface. These interactions lead to a new “biological identity” for the nanoparticles and new physico-chemical and biological properties [1,2,3,4]. Thus, in direct agreement with literature reports [6,15,16,17,18,19,20,21,22,23,24,25,26,27,28,29,30,31,32], the present findings suggest that the entity of interest after interaction with the plasma proteins is not the nanoparticle itself. Instead, the particle and its ‘corona’ are adsorbed plasma proteins [6,15,16,17,18,19,20,21,22,23,24,25,26,27,28,29,30,31,32].

Albumin is an essential blood protein present in significant concentrations in human blood plasma. It is commonly used to study drug interactions and as a carrier for various active pharmaceutical ingredients [33]. Despite the abundant research in the field, the mechanisms of protein corona formation are not fully understood, particularly for complex NPs. Some studies have shown that iron oxide (Fe_3_O_4_) NPs spontaneously interact with albumin and other proteins, depending on the properties of the NPs and proteins. Thus, we aimed to investigate the influence of adding multiple functional layers on the surface of IONPs and their interaction with albumin. The surface structure of coated NPs can correspond to different levels of albumin affinity, potentially leading to differences in thermodynamic parameters (ΔHo, ΔSo, and ΔGo) and binding forces between the NPs and proteins [34]. Thus, we investigated the binding properties of each nanostructure presented to albumin to understand the influence of NP coatings on protein corona formation.

## 2. Materials and Methods

Human serum albumin (HSA, lyophilized powder, fatty acid-free), sodium azide, iron(III) chloride hexahydrate, and iron(II) chloride tetrahydrate were purchased from Merck KgaA (Darmstadt, Germany). *Uncaria tomentosa* L. bark, *Clinopodium vulgare* L. aerial parts, and *Ganoderma lingzhi* mushroom were purchased from a local pharmacy. All other reagents were of analytical grade. HSA stock solution (3 mg/mL) was prepared by dissolving HSA in distilled water containing 0.01% sodium azide and stored at 0 ÷ 4 °C.

### 2.1. Preparation of Plant Extracts

Aqueous plant extracts were prepared as follows: 1 g of dry plant material was added to a beaker with 100 mL of distilled water preheated to 80 °C. The mixture was stirred with an electromagnetic stirrer for 60 min, maintaining the temperature. The resulting aqueous extracts were separated by filtration through a 0.45 μm pore size membrane filter under vacuum and were immediately used for NP synthesis.

### 2.2. Green Synthesis of Iron Oxide Nanoparticles

First, FeCl_3_·6H_2_O and FeCl_2_·4H_2_O were dissolved in distilled water in a molar ratio of 2:1. To 200 mL of this solution, 50 mL of each aqueous plant extract was added, and the mixture was stirred for 30 min at 80 °C under a nitrogen atmosphere. During the process, pH was maintained above 6 by adding NaOH. The obtained nanoparticles were separated with a magnet and washed three times with distilled water. An IONP aqueous suspension at a concentration of 3 mg/mL was used for spectroscopy studies.

### 2.3. Characterisation of Iron Oxide Nanoparticles

The synthesized nanoparticles were characterized in terms of physical and chemical properties that may influence their interaction with albumin. Fourier transform infrared spectroscopy (FTIR) was performed using a Nicolet iS 10 FTIR 189 spectrometer (Thermo Fisher Scientific, Pittsburgh, PA, USA) for functional group determination. The spectra were collected in the range of 400–4000 cm^−1^ and visualized with the OMNIC^®^ software package (Version 7.3, Thermo Electron Corporation, Madison, WI, USA). The surface morphology was investigated using a Prisma E model (Thermo Scientific, Waltham, MA, USA) scanning electron microscope (SEM). Energy -dispersive X-ray analysis (EDX) was performed alongside the SEM to create elemental maps of the samples. Dynamic light scattering (DLS) was applied to determine the particle size and zeta potential of the nanosized structures using a Zetasizer Pro (Malvern Panalytical Ltd., London, UK).

### 2.4. Fluorescence Spectroscopy

A FluoroMate FS-2 spectrometer (SCINCO CO. Ltd., Seoul, Republic of Korea) was used to measure fluorescence spectra using a 1.0 cm quartz cuvette. The excitation and emission bandwidths were set to 5 nm. Fluorescence measurements of the IONPs-HSA complex were performed, with the concentration of HSA fixed at 0.3 mg/mL and that of the IONPs ranging from 0.15 to 1.05 μg/mL. Fluorescence spectra were recorded after a 30-min incubation of each sample at three different temperatures (15, 25, and 37 °C) in the emission spectral range 310 ÷ 500 nm under excitation at 286 nm (n = 10 replicates).

### 2.5. UV-Vis Absorption Spectroscopy

UV-Vis absorption spectra were obtained on a Shimadzu UV 1601 spectrophotometer (Shimadzu Corporation, Kyoto, Japan) with a 1.0 cm path length cell. Samples of HSA (2.3 mg/mL), in the presence and absence of IONP@UT, IONP@CV, and IONP@GL (10.5 μg/mL) in distilled water, were incubated for 30 min, and spectra were measured in the range 190 ÷ 350 nm (*n* = 10 replicates) at room temperature.

## 3. Results and Discussion

### 3.1. Characterisation of Iron Oxide Nanoparticles

Iron oxide nanoparticles were obtained by green synthesis (reduction of iron chloride precursor by plant extracts). The formation of a black precipitate indicated that IONPs were produced, confirming numerous reports in the literature [35,36,37,38,39,40,41,42,43,44].

#### 3.1.1. Fourier Transform Infrared Spectroscopy (FTIR)

**Figure 1:**
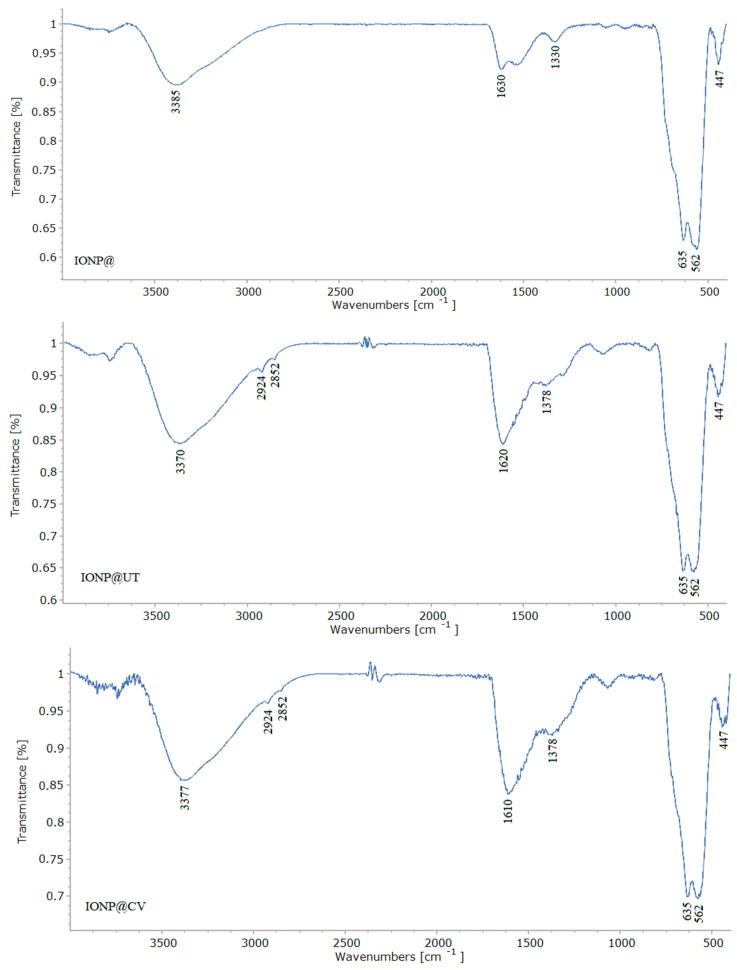

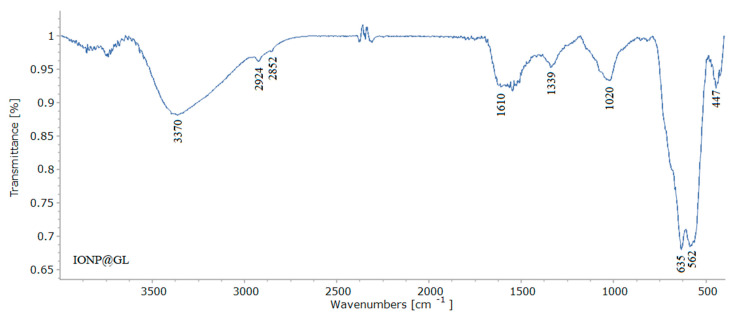
FTIR spectra of iron oxide nanoparticles without bioactive corona (IONP@) and samples obtained by green synthesis using aqueous extracts from Uncaria tomentosa (IONP@UT), Clinopodium vulgare (IONP@CV), and Ganoderma lingzhi (IONP@GL).

FTIR analysis was used to investigate the composition of the fabricated NPs. The generated spectra are presented in Figure 1. The absorption peak observed at approximately 3370 cm^−1^ and 1625 cm^−1^ corresponds to the stretching vibrations of OH groups from water. The infrared bands below 700 cm^−1^ are attributed to Fe–O bond vibrations, characteristic of iron oxide [35]. The peaks at 2924 cm^−1^ and 2852 cm^−1^ are associated with CH_2_ vibrations, while the peak at 1378 cm^−1^ is attributed to CH_2_ or CH_3_ vibrations. Additionally, the peak around 1030 cm^−1^ in the spectrum of IONP@GL indicates the presence of the chitin-glucan complex [45,46].

#### 3.1.2. Morphological Structure

**Figure 2:**
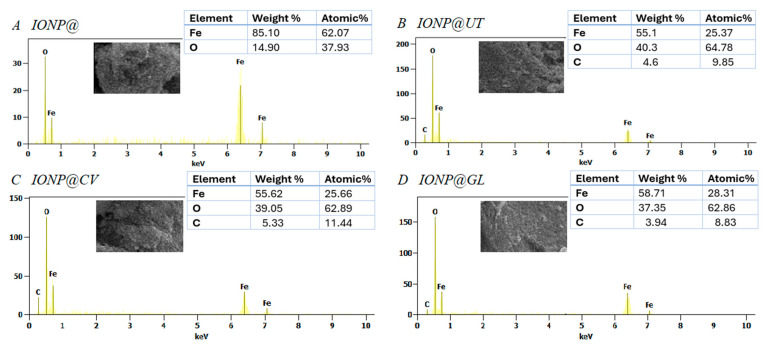
SEM and EDX analysis of iron oxide nanoparticles without Bioactive corona (**A**) and samples obtained by green synthesis using aqueous extracts from *Uncaria tomentosa* (**B**), *Clinopodium vulgare* (**C**), and *Ganoderma lingzhi* (**D**).

Scanning electron microscopy with an energy -dispersive spectroscopy detector was used to explore the elemental composition of the IONPs and validate the presence of an organic coating on the NPs. The EDX spectra (Figure 2) showed that the uncoated IONP@ sample is composed of Fe and O, with characteristic peaks for Fe Lα (0.66 keV), Fe Kα (6.4 keV), Fe Kβ (7 keV), and O Kα (0.52 keV). The IONP@ sample (Figure 2A) exhibited a higher Fe-to-O atomic ratio compared to theoretical values (0.75 for Fe_3_O_4_ and 0.667 for Fe_2_O_3_, etc.), suggesting the possible presence of pure Fe atoms in the sample. In the spectra of the coated NPs (Figure 2B–D), a carbon peak was detected, indicating the formation of an organic shell around the NPs. SEM images showed the formation of flake-shaped nanoparticles with a tendency for agglomeration, as confirmed by DLS analysis.

#### 3.1.3. Particle Size Distribution and Zeta Potential

**Figure 3:**
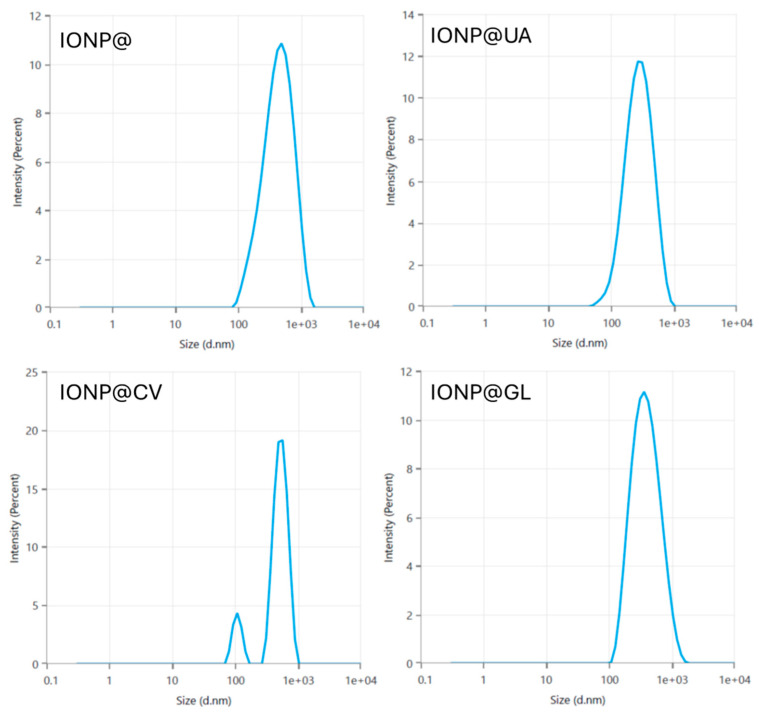
DLS distribution curves of nanoparticles of differen batches.

Dynamic light scattering was used to determine the hydrodynamic diameter, zeta potential, and polydispersity indices of green -synthesized nanoparticles. The results provided evidence that structures with an average hydrodynamic diameter in the range of 250 nm to 533 nm were fabricated. The polydispersity index had low values, indicating a homogeneous particle size distribution, except for the batch IONP@CV. The DLS curve of the latter had two distinct peaks, suggesting the formation of aggregates due to low thermodynamic stability, which correlates to the low zeta potential value measured for IONP@CV. The zeta potential appeared to be positive for IONP@ and turned negative for corona-coated NPs, indicating the formation of a corona of bioactive molecules around the iron oxide core. High zeta potential values were measured for IONP@GL and IONP@, suggesting increased long-term stability of the nanoparticles. The lowest zeta potential value was found for IONP@CV, indicating low surface stability and agglomerate formation. This finding is confirmed by the DLS distribution curve obtained for this sample. The obtained data are presented in Table 1, and particle size distribution curves are shown in Figure 3.

### 3.2. Fluorescent Spectroscopy

#### 3.2.1. Binding of IONPs to Human Serum Albumin

In general, fluorescence quenching refers to a process that reduces the fluorescence intensity of a fluorophore. It is well known that the intrinsic fluorescence of HSA is due to the presence of specific amino acids, mainly tryptophan (Trp) and tyrosine (Tyr) residues [8]. Ligand binding to the protein can directly affect the fluorescence of the tryptophan residue by acting as a quencher or by interacting with the fluorophore, dynamically changing the polarity of the surrounding conditions. Therefore, fluorescence quenching is a suitable technique based on modifying the intrinsic fluorescence of tryptophan in a non-destructive manner [47]. The interaction of the fluorophore with drugs [48] or NPs [49] modifies the amino acid environment, resulting in significant quenching.

#### 3.2.2. Correction of the Inner Filter Effect

The instrumental inner filter effect would cause some decrease in the fluorescence emission intensity with increasing concentrations of the drugs. This effect is an inherent problem of many fluorometric procedures, which can lead to results departing from their initial linearity and, therefore, must be considered. To avoid the inner filter effect caused by the adsorption of exciting light and reabsorption of the emitted light, we calculated the sum of absorbance at the excitation wavelength (286 nm) and the emission wavelength (about 337 nm) under different conditions to eliminate the effect. The following equation was used to correct the inner filter effect in the fluorescence data [50]:

(1) $$F_{cor}=F_{obs}e\;\frac{A_{ex}+A_{em}}{2}$$

where F_cor_ and F_obs_ are the corrected and observed fluorescence intensity, respectively, and A_ex_ and A_em_ are the sample absorbances at the excitation (286 nm) and emission (310 ÷ 500 nm), respectively.

#### 3.2.3. Fluorescence Quenching Method

**Figure 4:**
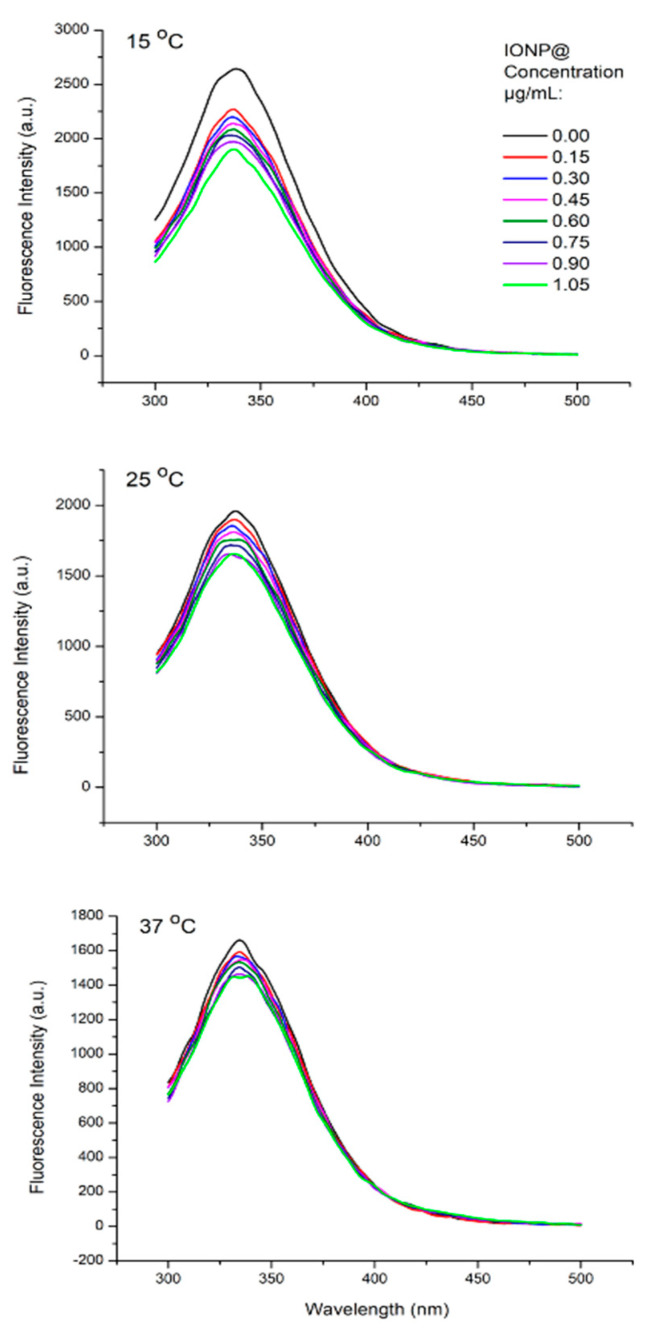
Fluorescence spectra of an HSA solution (0.3 mg/mL) before and after titration with naked nanoparticles IONP@ at 15, 25, and 37 °C. Excitation wavelength: 286 ± 1 nm.

**Figure 5:**
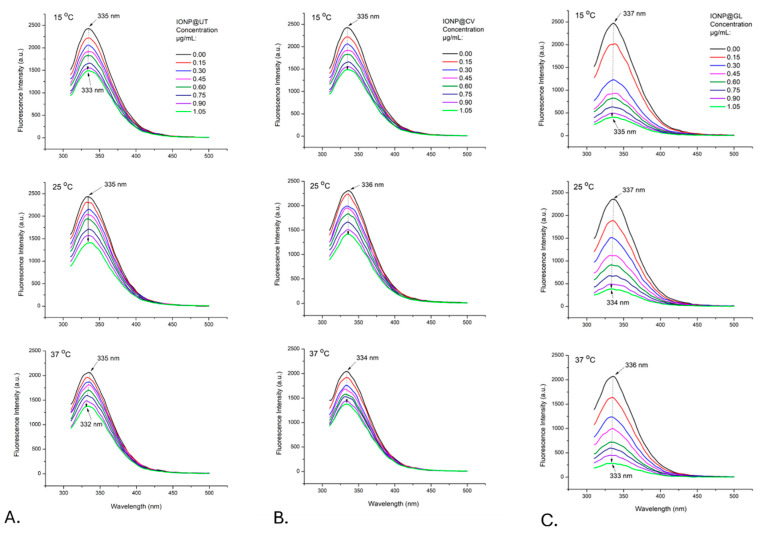
Fluorescence spectra of HSA solution (0.3 mg/mL) before and after titration with IONP@UT (**A**), IONP@CV (**B**), and IONP@GL (**C**) at 15, 25, and 37 °C. Excitation wavelength: 286 ± 1 nm.

Fluorescence spectra of HSA in distilled water were recorded in the absence and presence of the naked and the three types of iron oxide nanoparticles—IONP@UT, IONP@CV, and IONP@GL. HSA exhibited one excitation wavelength (λ_ex_) at 286 ± 1 nm and one emission wavelength (λ_em_) at 336 nm ± 1 nm. All investigated types of NPs without albumin showed negligible fluorescence. Upon addition of NPs with increasing concentration, the fluorescence intensity of albumin decreased at the same λ_em_. The fluorescence quenching measured at three different temperatures (15, 25, and 37 °C) indicated that an interaction between HSA and all the types of IONPs occurred (Figure 4 and Figure 5A–C)).

The results show that the fluorescence quenching is more substantial for the nanoparticles coated with the three natural substances (IONP@UT, IONP@CV, and IONP@GL) than the quenching observed for the naked nanoparticles. This shows us that coating contributes to a better interaction of nanoparticles with albumin compared to its interaction with naked nanoparticles. Furthermore, the results show a blue shift of the maximum spectra up to 6 nm observed after increasing the concentration of IONP@CV and IONP@GL in the investigated mixtures. This reveals an increase in hydrophobic amino acid residues in the fluorophore microenvironment of the HSA molecule in these solutions [51,52]. Accordingly, these results indicate that the interaction between IONP@CV, resp. IONP@GL, with HSA, forms more hydrophobic amino acid groups.

The following crucial experimental step was to determine the nature of the complex between the studied NPs and albumin molecules since their interaction may be either static or dynamic. The type of interactions was investigated using the Stern-Volmer equation:

(2) $$\frac{F_{0}}{F}=1+K_{SV}\left[Q\right]=1+K_{q}\;\tau_{0}$$

Respectively, F is the fluorescence intensities in the absence and presence of a quencher; K_q_ is the bimolecular quenching constant; τ_0_ is the lifetime of the fluorophore in the absence of a quencher; Q is the concentration of the quencher; and K_SV_ = K_q_/τ_0_ is the Stern-Volmer quenching constant [53].

**Figure 6:**
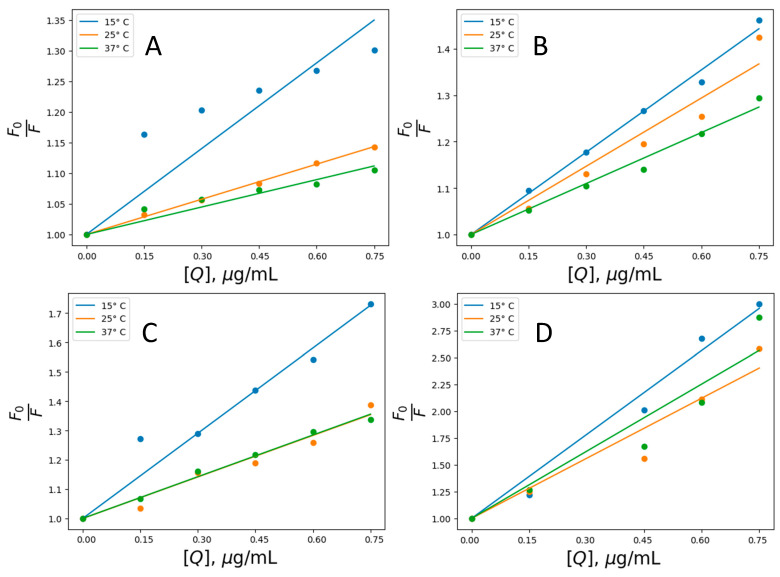
Stern–Volmer plot for the binding of IONP@ (**A**), IONP@UT (**B**), IONP@CV (**C**) and IONP@GL (**D**) nanoparticles with HSA at 15, 25, and 37 °C, (HSA) = 0.3 mg/mL.

Figure 6 presents the quenching data as plots of F_0_/F versus [Q]. The ratio F_0_/F is expected to depend linearly on the concentration of the quencher. The slope of the plot of F_0_/F versus [Q] yields an intercept of one on the y-axis, giving the K_SV_ value. To obtain values for the fluorescent quenching constant, we used the Nedler-Mead simplex algorithm to fit a linear regression model to F_0_/F and Q for the values of studied NPs in the range 0 ÷ 0.75 μg/mL.

Dynamic or static quenching is determined by the dependence of the fluorescence maximum on temperature and viscosity [53]. Plots of F_0_/F vs. [Q] (Figure 6) indicate the types of quenching: a straight line for individual static or dynamic quenching and an upward curve for mixed types. Static quenching is often observed if the fluorophore and the quencher can have a stacking interaction [53].

Table 2 presents binding parameters estimated for IONP@UT, IONP@CV, and IONP@GL fitted according to the Stern-Volmer equation from Figure 4. The parameters show an inverse relationship between temperature and the calculated K_SV_ values. This indicates that the quenching mechanism observed in the IONPs-HSA binding reaction is likely initiated by ground -state stable complex formation rather than dynamic collision [54,55]. Calculating the quenching rate constant (K_q_) further corroborates evidence favoring the quenching mechanism [54,55]. It is generally considered that the maximum scatter collision quenching of different quenchers for dynamic quenching is 2 × 10^10^ M^−1^s^−1^. The data presented in Table 2 show that the calculated quenching rate constants in the present case are orders of magnitude higher than the maximum threshold for the scattering collision quenching constant, justifying the dominant role of complex formation in the observed quenching process in the investigated system [54,55,56].

#### 3.2.4. Determining the Type of IONPs-HSA Interaction

The interaction forces between nanoparticles and biomolecules can be diverse, including electrostatic, hydrogen bonding, van der Waals interaction, hydrophobic and steric contact at the binding site, etc. [54,55,57,58,59,60]. The signs and magnitudes of thermodynamic parameters for the binding reaction between proteins and nanoparticles are often used to explain the underlying binding forces [54,55,57,58,59,60].

**Figure 7:**
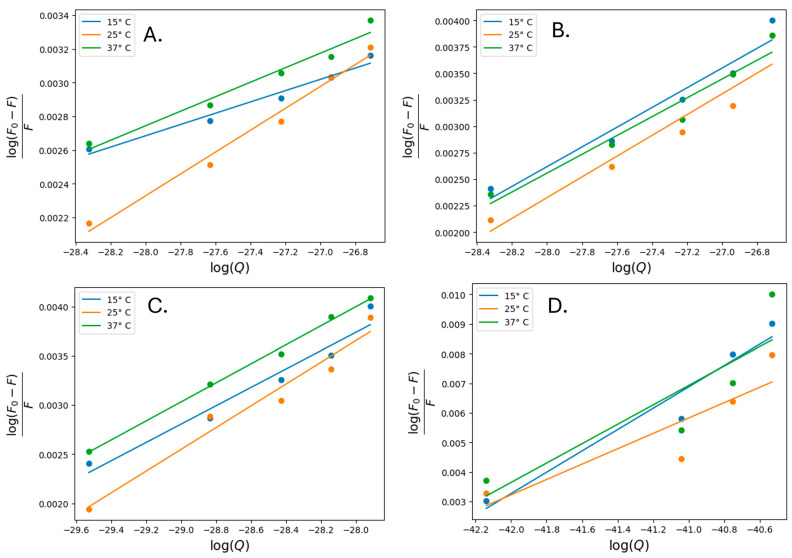
Double logarithmic plots of the interaction between HSA and IONP@ (**A**), IONP@UT (**B**), IONP@CV (**C**), and IONP@GL (**D**) nanoparticles at 15, 25, and 37 °C.

A quantitative assessment of the interaction between IONPs and HSA can be derived from the estimation of the binding constant (K_a_) and the thermodynamic parameters of the change in enthalpy (ΔH°), entropy (ΔS°), and free energy (ΔG°) for the IONPs–protein complexation equilibrium process. The calculation of the binding parameters from the fluorescence quenching data is based on the following formulas widely used in the literature [27,28,30,31,54,55,61,62]:

(3) $$\frac{\log\left(F_{0\;}-F\right)}{F}=\log K_{a}+n\;\log\left[Q\right]$$

(4) $$\;K_{q}=\frac{K_{SV}}{\tau_{0}}$$

(5) $$\Delta G^{{}^{\circ}}=\Delta H^{{}^{\circ}}-T\Delta S^{{}^{\circ}}=-RT\ln K_{a}$$

(6) $$\ln K_{a}=-\frac{\Delta H^{{}^{\circ}}}{RT}+\frac{\Delta S^{{}^{\circ}}}{T}$$

where Ka, n, ΔH°, ΔS°, ΔG°, T, and R are the binding constant, the number of binding sites, enthalpy change, entropy change, Gibbs free energy change, the temperature (15, 25, and 37 °C), and the gas constant, respectively. The thermodynamic parameters for IONPs-HSA complexes were calculated using Equations (3)–(6) and the Wan’t Hoff plot (Figure 6). The data are shown in Table 3. The graphs in Figure 7 and the data presented in Table 3 show the results for the binding constant (Ka) and thermodynamic parameters (ΔH°, ΔS°, and ΔG°) of the IONPs–HSA complex. The negative value for ΔH° indicates an exothermic binding reaction in which energy is released into the environment. The derived thermodynamic parameters presented in Table 3 suggest that the binding IONPs-HSA is entropy favored (ΔS° > 0), with an overall negative Gibbs free energy change suggesting spontaneity of the interaction occurrence [54,55,63,64,65]. Furthermore, the negative enthalpy value and positive entropy change indicate that the binding process between HSA and IONPs is dominated by electrostatic interactions [63,64,65].

Understanding the interaction between proteins and NPs is fundamental for predicting therapeutic responses. In this work, we investigated the mechanisms of interaction between albumin and NPs that lead to the formation of a protein layer. Structural changes in the protein chain after interaction with NPs can positively or negatively affect the fate of NPs in vivo in terms of toxicity and cellular uptake [66].

### 3.3. UV-Vis Analysis of the Interaction Between HSA and IONPs

**Figure 8:**
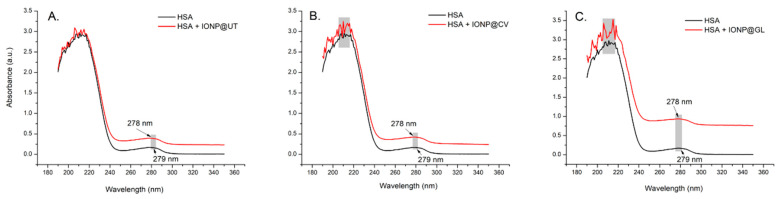
Absorbance spectra of HSA and IONP@UT (**A**), IONP@CV-HSA (**B**), and IONP@GL-HSA (**C**) complexes. HSA concentration was 2.3 mg/mL, and the IONPs concentration for the IONPs-HSA complex was 10.5 μg/mL.

UV-Vis spectroscopy provides preliminary information on the interaction profile of ligands with proteins. In addition, it identifies possible perturbations in the secondary structure of biomacromolecules [67]. Another method to distinguish between static and dynamic quenching was performed by examining the UV spectra of HSA, IONPs, and IONPs-HSA mixtures. Figure 8 shows the obtained UV-Vis spectra.

The figure displays two absorption peaks: one at 222 nm and another at 279 nm, corresponding to π→π* and *n*→π* transitions, respectively of the aromatic amino acid residues tryptophan (Trp), phenylalanine (Phe) and tyrosine (Tyr) [68], while IONPs did not show any significant UV-Vis absorption peaks. A slight hyperchromic effect was observed in the range 267 ÷ 300 nm, probably due to the interaction of HSA and IONPs in the ground state (a static mechanism) [69], indicating that the NPs interact weakly with HSA under the conditions studied, possibly because of the chemical structure and high solubility of ligands in aqueous medium [70]. The similar UV-Vis spectral profile in the range 190 ÷ 250 nm for HSA without and in the presence of NPs suggests that the ligands cause a weak perturbation of albumin’s secondary structure [71]. It can be assumed that the absorption spectra of individual HSA fluorophores and quenching mixtures are perturbed. In contrast, it was suggested that no changes in the absorption spectra occur during dynamic quenching [68]. From the obtained Stern–Volmer plots and UV-Vis spectra, we can assume that the fluorophores of HSA were quenched using IONPs by collision and stable complex formation [72,73,74].

## 4. Conclusions

The present study shows that the interaction between HSA and NPs depends on the nanoparticle structure. The binding between IONPs and HSA was found to be spontaneous. The interaction is exothermic and entropy-driven; ionic interactions play a leading role. HSA interacts weakly with IONP@, moderately with IONP@UT and IONP@CV nanoparticles, and firmly with IONP@GL nanoparticles through ground-state association. Analysis by a modified Stern-Volmer approximation indicates that the quenching mechanism is static. The NP–protein association is frequently driven by weak interactions, and its mechanism enables each particle’s surface stabilization [75]. In our study, the naked and the three NPs have different affinity constants (K_a_) values, weak for naked IONP@, and coated IONP@UT, and IONP@CV nanoparticles and high for IONP@GL, probably due to the formation of the varying protein corona. Thus, it is assumed that the coating of the NPs with plant extract leads to an increased affinity for a particular protein in a biological fluid, the highest effect observed for IONP@GL, and affects the physicochemical characteristics, subsequently affecting the rate and extent of biodistribution.

This study contributes to a better understanding of the interaction, distribution, and transport mechanisms involved in protein-NP interactions by providing insights into functional perturbations of albumin’s binding capacity.

## Figures and Tables

**Table 1:** Hydrodynamic particle size, polydispersity index (PDI) and zeta potential of nanoparticles of different batches.

NPs Type	Particle Size [nm]	PDI	Zeta Potential [mV]
IONP@	374	0.216	41.30
IONP@UT	250	0.208	−20.67
IONP@CV	533	0.611	−17.29
IONP@GL	332	0.239	−77.89

**Table 2:** Stern-Volmer quenching constants (K_SV_) and quenching range constant (K_q_) for IONPs-HSA complexes with thermodynamic parameters (ΔH°, ΔS°, and ΔG°) for the interaction of HSA with NPs at 15, 25, and 37 °C.

NPs Type	T [°C]	K_sv_ [M^−1^]	R^2^	Kq [M^−1^s^−1^]
	15	14.00 × 10^4^	0.722	14.00 × 10^12^
IONP@	25	5.70 × 10^4^	0.998	5.70 × 10^12^
	37	4.50 × 10^4^	0.897	4.50 × 10^12^
	15	17.70 × 10^4^	0.992	17.70 × 10^12^
IONP@UT	25	14.70 × 10^4^	0.944	14.70 × 10^12^
	37	11.00 × 10^4^	0.970	11.00 × 10^12^
	15	29.10 × 10^4^	0.945	29.10 × 10^12^
IONP@CV	25	14.20 × 10^4^	0.962	14.20 × 10^12^
	37	14.20 × 10^4^	0.990	14.20 × 10^12^
	15	10.89 × 10^5^	0.971	10.89 × 10^13^
IONP@GL	25	8.98 × 10^5^	0.925	8.98 × 10^13^
	37	8.65 × 10^5^	0.948	8.65 × 10^13^

**Table 3:** Binding constant (Ka) and thermodynamic parameters (ΔH°, ΔS°, and ΔG°) for the interaction between HSA and IONP@, IONP@UT, IONP@CV, and IONP@GL nanoparticles at 15, 25, and 37 °C.

NPs	T [°C]	K_a_ [M^−1^]	R^2^	ΔH° [kJmol^−1^]	ΔS° [Jmol^−1^]	ΔG° [kJmol^−1^]
IONP@	15	2.74 × 10^2^	0.996	−25.70	207.80	−13.44
	25	5.24 × 10^2^	0.987		207.80	−15.51
	37	3.50 × 10^2^	0.985		198.40	−15.10
IONP@UT	15	7.75 × 10^2^	0.991	−6.75	41.79	−18.78
	25	8.21 × 10^2^	0.976		43.09	−19.59
	37	7.41 × 10^2^	0.993		43.09	−20.10
IONP@CV	15	6.94 × 10^2^	0.953	−6.74	41.81	−18.79
	25	8.78 × 10^2^	0.931		43.71	−19.78
	37	7.63 × 10^2^	0.954		43.43	−20.21
IONP@GL	15	3.10 × 10^9^	0.989	−5.56	108.75	−22.87
	25	2.35 × 10^9^	0.929		107.12	−23.47
	37	3.00 × 10^9^	0.906		107.12	−24.76

## Data Availability

Data is contained within the article.
